# Molecular Mechanisms of Reconsolidation-Dependent Memory Updating

**DOI:** 10.3390/ijms21186580

**Published:** 2020-09-09

**Authors:** Lauren Bellfy, Janine L. Kwapis

**Affiliations:** Department of Biology, Center for Molecular Investigation of Neurological Disorders (CMIND), Pennsylvania State University, University Park, PA 16802, USA; lyb5150@psu.edu

**Keywords:** updating, memory, reconsolidation, destabilization, restabilization

## Abstract

Memory is not a stable record of experience, but instead is an ongoing process that allows existing memories to be modified with new information through a reconsolidation-dependent updating process. For a previously stable memory to be updated, the memory must first become labile through a process called destabilization. Destabilization is a protein degradation-dependent process that occurs when new information is presented. Following destabilization, a memory becomes stable again through a protein synthesis-dependent process called restabilization. Much work remains to fully characterize the mechanisms that underlie both destabilization and subsequent restabilization, however. In this article, we briefly review the discovery of reconsolidation as a potential mechanism for memory updating. We then discuss the behavioral paradigms that have been used to identify the molecular mechanisms of reconsolidation-dependent memory updating. Finally, we outline what is known about the molecular mechanisms that support the memory updating process. Understanding the molecular mechanisms underlying reconsolidation-dependent memory updating is an important step toward leveraging this process in a therapeutic setting to modify maladaptive memories and to improve memory when it fails.

## 1. Introduction

Memories are not stable records of experience but are flexible and dynamic entities that can be modified or updated to maintain relevance in the face of changing information. Reconsolidation-dependent memory updating is a process that guides behavior [1,2,3] and either strengthens an existing memory and the associated behavior [1,4] or weakens/modifies the original memory, resulting in modified behavior [3]. This process is an attractive method for potentially treating a range of psychiatric disorders that stem from maladaptive memories, as reconsolidation-dependent updating could be used to change the content or emotional valence of traumatic, aversive, or problematic memories. Indeed, in a laboratory setting, animal models of both post-traumatic stress disorder (PTSD) [5,6,7,8] and addiction [9,10,11,12,13,14] have shown improvements following updating-based procedures, suggesting that this process could be transitioned into the clinical setting [3,4]. Understanding the molecular and cellular mechanisms that support reconsolidation-dependent memory updating is, therefore, a key step toward improving treatments for PTSD and other disorders that stem from problematic associations. 

When a new memory is formed, it must initially go through the consolidation process before becoming a stable long-term memory [15,16,17]. During this transient period (typically lasting a few hours after acquisition), the memory is temporarily labile, susceptible to disruption from any number of amnesic treatments, such as protein synthesis inhibitors [15]. Once consolidated, the stored memory is largely resistant to disruption by these amnesic agents [17] until it is retrieved. When the animal is given a reminder of the training session [18], the memory temporarily becomes labile again before being restabilized, a process termed “reconsolidation” [1,2,3,15,19]. Although the purpose of this reconsolidation process is not entirely clear, recent work has suggested that reconsolidation functions in part to allow existing memories to be updated in response to new information [18,20,21,22,23,24].

In this review, we will cover the discovery and importance of reconsolidation, describe the major behavioral paradigms used to investigate reconsolidation-dependent memory updating in the laboratory, and outline the molecular mechanisms that support this updating process. While the cellular and molecular mechanisms that support memory updating are just starting to be elucidated, massive strides have been made in recent years with the development of new technical and behavioral tools, including our new paradigm called the objects in updated locations (OUL) task.

## 2. Rediscovery of Reconsolidation

**Figure 1:**
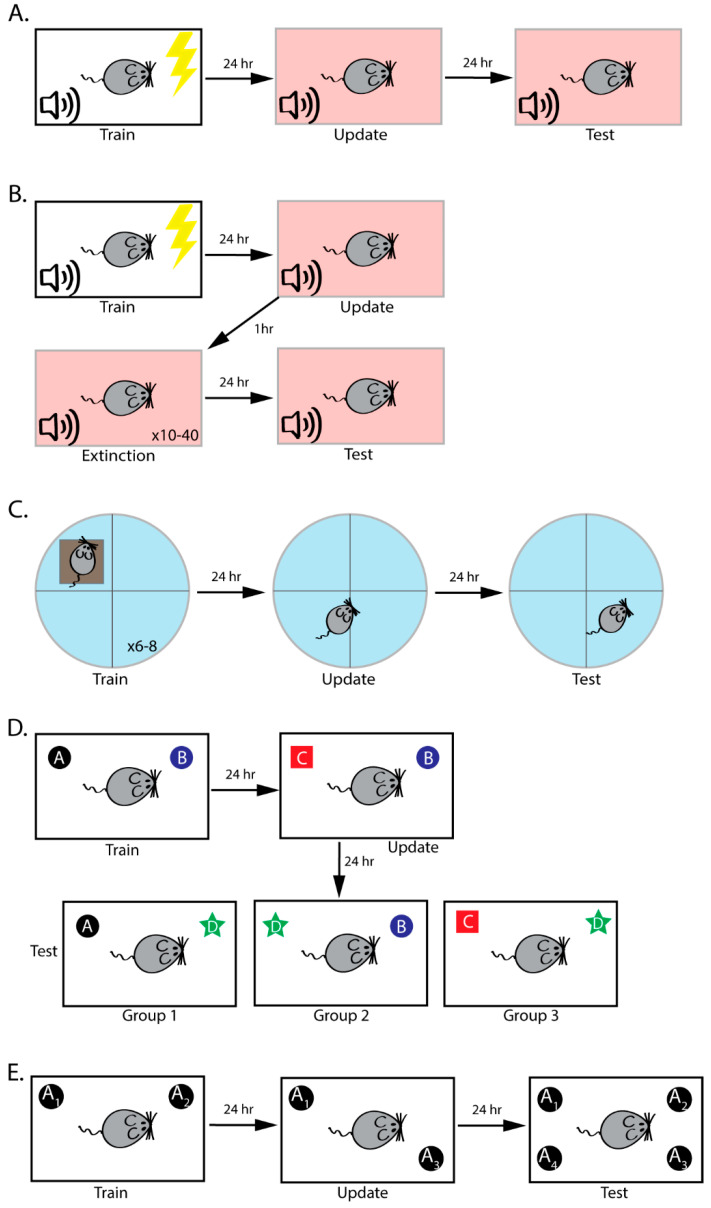
Schematics of common paradigms used to study reconsolidation-dependent memory updating. (**A**) A typical fear conditioning-reconsolidation experiment. Animals are initially trained to learn the CS-UCS association. Pictured, a tone CS is paired with a footshock UCS. Then, 24 hours later, animals are placed in a new context (peach background) and exposed to the CS to drive memory retrieval and updating. On the final day, the animal is presented with the auditory CS in the retrieval context. Throughout the experiment, freezing is measured as an index of fear. (**B**) Typical procedure for the reconsolidation-extinction paradigm. Training and retrieval sessions are identical to (A). One hour following retrieval, the animals are placed back into the retrieval context and the CS is presented multiple times to extinguish the fear memory. Extinction memory is tested the following day, with low freezing indicating successful formation of extinction memory. (**C**) A typical procedure for studying updating in the Morris water maze. Rodents are first trained to learn the location of a hidden platform in a round pool. The following day, the memory is updated during a probe test, with the platform removed. Memory is tested with a second probe test the following day, with the researcher measuring the percentage of time the animal spends in the target quadrant as an index of memory. (**D**) Schematic of the object recognition updating paradigm. During training, rodents learn the identities of two objects. The following day, this memory is updated by replacing one familiar object with a novel item. Memory for the original and updated memories is tested 24 h later by exposing separate groups of animals to one of the three familiar objects with a novel object. Preferential exploration of the novel object indicates the animal remembers the familiar item. (**E**) Schematic of the objects in updated locations (OUL) paradigm. During training, rodents are exposed to two copies of an object in specific locations (A_1_ and A_2_). Memory is then updated by moving one object to a new location (A_3_). The following day, memory is tested by exposing the animal to four objects: three in familiar locations (A_1_, A_2_, and A_3_) and one in a novel location (A_4_). Memory for the original locations (A_1_ and A_2_) and the updated location (A_3_) can be assessed by comparing exploration of each familiar location to exploration of the novel location A_4_.

Reconsolidation was first discovered in 1968 [25], but was essentially forgotten until 2000 when it was “rediscovered” by Nader and colleagues using fear conditioning. In fear conditioning, an emotionally neutral conditional stimulus (CS), such as a tone is paired with a naturally aversive unconditional stimulus (UCS), usually a foot shock. Rodents rapidly learn this association, so that future presentation of the CS alone evokes a fear response, typically measured as increased freezing behavior. In the seminal Nader (2000) study, 24 h after fear-conditioning acquisition, rats were placed in a novel context and presented with the CS alone to drive memory retrieval. Immediately after the retrieval session, the rats received infusions of the protein synthesis inhibitor anisomycin (or vehicle) into the basolateral nucleus (BLA) of the amygdala, a region critical for fear memory. The following day, rats were tested for fear to the tone CS (Figure 1A). Anisomycin disrupted fear memory *only* if the animals were given a retrieval session, indicating that a stable, stored fear memory can be rendered labile by a single retrieval trial [26]. This study challenged the traditional assumption that consolidated memories are stored in a fixed, permanent fashion, demonstrating instead that reactivating a fully consolidated memory can revert it to a labile, malleable state amenable to updating [27].

Much of the subsequent work on reconsolidation has been performed using fear conditioning, but in recent years, this work has been extended to multiple types of memory, including spatial, taste, and human episodic memory paradigms [1,16] (more on paradigms in Section 3). These paradigms have been used in many different species, from medaka fish [28] to honeybees [29,30], nematodes [31], snails [32], crabs [33], and chickens [34], with most of the work performed in mice [2,12,35,36,37,38,39,40,41,42,43,44,45,46,47,48,49,50,51], humans [52,53,54,55,56,57,58], and rats [6,8,9,13,14,19,20,21,22,23,24,25,26,28,59,60,61,62,63,64,65,66,67,68,69,70,71,72,73,74,75,76,77,78,79,80,81,82,83,84,85,86,87,88,89,90,91,92,93,94,95,96,97,98,99,100,101,102]. Across these different species and memory tasks, the major finding is the same: memory retrieval can make a consolidated memory labile and susceptible to reconsolidation-dependent memory updating.

There are limitations in each type of memory paradigm that preclude memory reconsolidation. These limitations, termed boundary conditions, include memory strength [16,19], memory age [1,4,16], extinction, and, most notably, the predictability of the reactivation stimulus [1,16]. Through the work of many groups, we now know that one critical requirement needed to trigger reconsolidation is the presentation of new information; when only familiar information is presented at retrieval, memory remains stable and resistant to amnesic agents [2,20,21,22,39,75,103]. It is only when new information is presented that reconsolidation is initiated, presumably to allow the content of that memory to change or update [18,19,21,22,75]. A context shift alone can drive reconsolidation-dependent memory updating [22], as can a change in stimulus timing or contingency [20,21]. Notably, in the Nader 2000 study, the retrieval session included at least two pieces of new information: the CS was presented in a new context and was no longer followed by the UCS, changing both the context and the contingency [26]. Studies have since shown that when a retrieval session does not include this new information (i.e., the familiar CS-UCS pairing is presented in the training context), reconsolidation does not occur [20,21,22]. In a clever application of reconsolidation-based updating, multiple groups have shown that a traumatic memory can be made less fearful by presenting an appetitive stimulus during the retrieval session, seemingly reducing the negative emotional valence of the original memory [58,65,68,84,85,104]. Finally, when looking at the brain at a circuit level, there is evidence that reconsolidation-based updating can be used to fundamentally reorganize the circuit that supports a memory [21,94]. 

## 3. Behavioral Paradigms to Study Reconsolidation-Dependent Memory Updating

### 3.1. Fear Conditioning

Fear conditioning is one of the most robust, well-characterized paradigms used to study reconsolidation. As previously mentioned, in fear conditioning, a neutral CS is paired with an aversive UCS and within a single pairing, the CS elicits a conditional fear response (CR) from the animal, such as freezing in rodents [15,16,20]. There are multiple variations of this paradigm, with auditory fear conditioning [20,22,47,48,61,72,80,92,100] and contextual fear conditioning being the most common (Table 1, [19,28,40,68,70,92]). 

There are many benefits to using fear conditioning to study reconsolidation-dependent memory updating. For one, the investigator has complete control over which stimuli are presented, the number of pairings, and the timing in which they are presented. Further, the learned behavior is robust, predictable, and easily measurable by quantifying the percentage of time the animal spends freezing [15]. Fear conditioning can also ethically be performed in humans, with great success [54,55,56], allowing for cross-species comparisons. Finally, fear conditioning is a good model for PTSD and other anxiety disorders [20,47], allowing us to understand the neural mechanisms associated with maladaptive memories. 

Despite the many advantages of using fear conditioning to understand reconsolidation-dependent memory updating, there are some important limits, or boundary conditions, that need to be considered. These boundary conditions can make it challenging to modify existing fear memories [2]. For example, a strong fear memory is usually resistant to updating [16,19]. Likewise, older memories are less likely to undergo reconsolidation-dependent memory updating [1,4,16]. Additionally, as freezing is not a carefully timed behavior, the researcher cannot easily determine whether a rodent is freezing in response to the original memory or the updated memory; fear conditioning cannot assess both memories in a single session [2]. Overall, however, fear conditioning has been invaluable in identifying the basic cellular and molecular mechanisms that support reconsolidation-dependent memory updating.

### 3.2. Reconsolidation-Extinction

Extinction occurs when the CS is presented numerous times without the UCS, reducing the association between the two stimuli [15,80]. Extinction does not produce erasure of the original fear association, instead creating a new, competing memory. This can ultimately lead to reemergence of the original fear memory, as can be demonstrated in the laboratory by measuring reinstatement [110], renewal [105], or spontaneous recovery [111]. Although it is clear that extinction involves new learning, it is also likely that the original fear memory is updated in response to extinction learning. One important observation is that conditional responding only partially recovers during spontaneous recovery, renewal, or reinstatement [112]. Further, extinction induces synaptic changes that resemble depotentiation, suggesting that the synaptic connections supporting the fear memory are weakened following extinction learning [113,114]. Thus, it is likely that extinction represents, at least in part, some updating of the original memory content in addition to the new learning of the CS-no UCS contingency.

In 2009, Monfils and colleagues developed an interesting reconsolidation-extinction paradigm that aims to eliminate the reemergence of the fear memory by capitalizing on the transient period of lability following memory retrieval [80]. The authors first destabilized an existing fear memory by presenting a retrieval trial and then, while the fear memory was susceptible to disruption, extinguished the memory (Figure 1B). When compared to a traditional extinction protocol, the reconsolidation-extinction protocol promoted a more permanent extinction memory, with less reemergence of the original fear memory [53,73,80]. The reconsolidation-extinction protocol has been found to promote more permanent extinction in both animal [47,72,73,74,80,91] and human studies [53], suggesting that it might be used to treat clinical disorders such as PTSD [4]. Unfortunately, this method does not always persistently attenuate memories [46,52,69,77,108,109,115], with remote memories showing particular resistance [47], and even direct replications have failed in some cases (Table 1, [77,107]). In 2010, Schiller and colleagues published a study using the reconsolidation-extinction paradigm in humans to successfully attenuate fear memories [53]. In 2020, Chalkia and colleagues published two papers in direct response to the Schiller (2010) paper [52,107] that cast doubt on the effectiveness of reconsolidation-extinction in humans. The first publication reported that, in a direct replication of the original study, the authors were unable to replicate the results [52]. For the second publication, Chalkia and colleagues obtained the original datasets from the Schiller (2010) paper to replicate the analyses on the same datasets. While a majority of the statistics from the original paper were replicated, there were discrepancies, with the main extinction effect of the Schiller (2010) paper relying heavily on qualitative, potentially inappropriate participant exclusion criteria [107]. Therefore, it is not currently clear whether the reconsolidation-extinction paradigm is an effective method of enhancing extinction in human participants. For reconsolidation-updating to be an effective method of persistently attenuating fear, we need to better understand how this paradigm works at both the behavioral and molecular levels.

### 3.3. Morris Water Maze

The Morris water maze (MWM) is a well-established spatial memory task in which a rodent learns to “escape” a round pool by finding a platform hidden below the surface of the water [38,86,87,92]. Over a series of days, animals learn the platform’s location using spatial cues placed around the maze. As rodents learn the location of the hidden platform, they show reduced latency to escape both within a single day and across training days. On the final day, a probe test is typically performed to test the animal’s long-term memory for the location of the platform. In this probe test, the platform is removed and the experimenter measures the percentage of time the rodent spends in the target quadrant as an index of memory [116].

The water maze has been adapted in a number of ways to allow researchers to study reconsolidation-dependent memory updating. In the most common version of this task, the probe test serves as a new learning situation (i.e., the animal learns that the platform is no longer in the expected location). As expected, treatment with a protein synthesis inhibitor immediately after this probe test update disrupts the original memory (Figure 1C, Table 1, [50,87]). 

As a spatial task, the water maze relies heavily on the hippocampus [38,86], an accessible target for spatially-specific manipulations. It has also been extensively used in behavioral neuroscience, so parametric information is readily available for this task. Despite these advantages, there are some potential drawbacks to using the water maze to understand reconsolidation-dependent memory updating. For one, the water maze can be stressful for animals [38]. Stress hormones can modulate learning and confound the results of molecular studies if not carefully controlled [117]. In addition, extensive swimming activity is required for the animals to successfully find the platform. This also become a confounding variable, as less physically fit rodents may have difficulty finding the platform due to less efficient swimming. This exercise requirement makes the water maze challenging, and potentially not appropriate, for older animals [38].

### 3.4. Object Recognition Memory

Object recognition memory (ORM) tests an animal’s ability to recognize an object’s identity in a simple, incidental learning paradigm [44,89]. In a typical ORM task, rodents learn the identity of two objects (or two copies of the same object) during acquisition and memory is tested by replacing one familiar object with a novel item. As rodents are driven to explore novelty, animals that remember the identities of the original objects will preferentially explore the novel object [118]. Rossato and colleagues (2007) developed a clever reconsolidation-dependent updating paradigm based on ORM that allows the researcher to assess both the original memory and the updated information, albeit in separate groups of animals. In this task, animals are first exposed to two objects, A and B. On the second day, object A is replaced with a novel object, object C. On the third day, trained animals are split into three groups to separately test memory for objects A, B, and C, by comparing exploration of each object to that of a new object, object D (Figure 1D, Table 1). Notably, the researchers found that post-update infusions of anisomycin disrupted memory for both the original and updated objects *only* if new information was presented during the update session. When the researchers presented only familiar information by re-exposing animals to the training objects A and B, anisomycin injections had no observable effect [89]. This is consistent with the idea that new information is required to trigger the reconsolidation process; when only familiar information is presented, the memory is not made labile and is not susceptible to anisomycin infusions.

A major advantage of this ORM updating task is that it relies on incidental learning and is, therefore, neither appetitive nor aversive [24]. This allows the researcher to avoid potential confounding variables engaged by emotionally charged paradigms, such as the activation of the stress pathway. In addition, both the original memory and the updated memory can be independently assessed in this task, although it requires separate groups of animals [89]. The perirhinal cortex is thought to be the major brain system that supports ORM, but limited work has been performed in understanding this region [94,97] compared to other learning-related structures, such as the hippocampus and amygdala. Notably, although some versions of this task seem to rely on the hippocampus, hippocampal manipulations do not always have an impact on ORM [118]. 

### 3.5. Objects in Updated Locations

We have recently developed a new reconsolidation-dependent memory updating paradigm called the objects in updated locations task, or OUL, that is based on the spatial object location memory task [2,41]. In OUL, the animal is first exposed to two copies of an identical object in specific locations in the training arena (A_1_ and A_2_), creating the original memory. On the second day, one of the objects is moved to a novel location (A_3_), updating the original memory with a new location. Finally, the original memory and the updated memory can be tested in a single session by placing a total of four objects in the box: two in the original locations (A_1_ and A_2_), a third in the updated location (A_3_), and a fourth in a novel location (A_4_; Figure 1E, Table 1). As mice prefer novelty, a mouse that remembers the original and updated object locations should preferentially explore the object in the novel location (A_4_) at test. Thus, by comparing exploration of the novel location to exploration of the other three locations, the researcher can infer whether the animal remembers the original and updated training locations [41].

We have used two different approaches to demonstrate that OUL engages and updates the original memory, rather than driving the formation of a new, independent memory. First, we injected anisomycin into the hippocampus following the update session to test whether the original object location memory was made labile in response to the update. Indeed, anisomycin impaired memory for both the original object location and the updated location, suggesting that the original memory was destabilized in response to the new object location, presumably to allow the existing memory to be updated. In a second experiment, we used *Arc* cellular compartment analysis of temporal activity by fluorescence in situ hybridization (catFISH) to test whether the update session reactivates the neuronal ensemble associated with the original memory. We observed a strong overlap between the neurons activated by the original memory and the update session. This indicates that the update session reactivates the neurons that support the original memory, rather than engaging a new set of neurons, as would likely occur if the update session was encoded as a separate, independent memory. [2].

One major advantage of OUL is that it can measure both an original memory and the updated information in a single test session. OUL is also hippocampus dependent, allowing the researcher to focus manipulations and molecular analyses on a specific, easily accessible brain region. Further, OUL is a non-emotional, low-stress testing paradigm that is appropriate for testing reconsolidation-dependent memory updating across the lifespan. We have successfully used OUL to detect age-related impairments in reconsolidation-dependent memory updating, demonstrating that although young mice (4–6 months of age) can easily remember the original and updated object locations, old mice (18–20 months of age) show updating impairments. Finally, OUL is also ideal for studying the molecular mechanisms uniquely engaged by reconsolidation-dependent memory updating, as the only difference between a memory update session and the original training session is the animal’s training history (i.e., whether the animal has already experienced one of those objects in a different location). The OUL paradigm, therefore, has a few advantages that can be leveraged to understand the cellular and molecular basis of reconsolidation-dependent memory updating [2,41].

## 4. Molecular Mechanisms of the Reconsolidation-Dependent Memory Updating Process

Work from a number of labs has begun to elucidate the molecular mechanisms that support reconsolidation-dependent memory updating (see Table 2), primarily relying on fear conditioning to identify key molecular players. It has become clear that the reconsolidation process consists of two phases: destabilization and restabilization. For memory to be updated, it must first be destabilized, or made malleable, in order to incorporate any new information presented during retrieval. Following the incorporation of new information, the memory undergoes a phase of restabilization before being placed back into storage [26,119,120]. Below, we will describe the molecular mechanisms known to support each phase of reconsolidation-dependent memory updating.

### 4.1. Molecular Mechanisms of Memory Destabilization

The destabilization phase is characterized by a period of deconstruction and protein degradation. If destabilization is prevented, the original memory is not rendered labile during retrieval. In this case, the memory is not affected by amnesic agents like protein synthesis inhibitors but is also not updated by the retrieval process. Instead, the memory will persist in its original state, without incorporating any new information [19,35,71].

One defining requirement for the destabilization phase is protein degradation through the ubiquitin-proteasome system (UPS). This period of protein breakdown is believed to drive memory lability by degrading existing synaptic connections, making the original memory malleable. Lee and colleagues (2008) first demonstrated this requirement, finding that memory retrieval drives an increase in poly-ubiquitination, a tag added to proteins to target them for degradation through the UPS. They further showed that a pharmacological inhibitor of degradation, βlac, prevents the memory-impairing effects of post-retrieval anisomycin, suggesting that protein synthesis is only necessary if a memory has first been destabilized through protein degradation [35]. In 2010, another researcher, Johnathan Lee, provided further support for the indispensable role of protein degradation in the destabilization process. Using the context pre-exposure facilitation effect (CPFE) paradigm, he cleverly separated the context learning phase from the subsequent context-shock association to demonstrate that protein degradation is necessary to update a neutral context memory with aversive content. Injecting βlac into the dorsal hippocampus following the immediate shock session (in which the animal learns to associate the existing context memory with an aversive footshock) disrupted freezing the following day, suggesting that these animals failed to incorporate information about the shock into the existing context memory [75]. This requirement for protein degradation in memory destabilization has since been replicated by a number of groups [71,121]. The field is currently working to understand *how* this increased protein degradation is triggered to destabilize an existing memory.

At the synapse, both AMPA and NMDA receptors are important for destabilization. AMPAR subunit composition seems to play a key role in determining whether synapses are malleable or whether they are stable and resistant to updating. At the most basic level, researchers have shown that post-retrieval injection of a general AMPAR inhibitor (e.g., CNQX or DNQX) prevents reconsolidation-dependent memory updating [31], indicating AMPARs are required for the destabilization process, but Mamou and colleagues (2006) found that a non-specific AMPAR inhibitor did not inhibit destabilization [99]. Calcium-permeable AMPA receptors (CP-AMPARs) in particular play an important role in destabilization. CP-AMPARs tend to be less stable than calcium-impermeable AMPARs (CI-AMPARs), as they lack the GluA2 subunit that regulates calcium to flow into the neuron [98]. This inherent instability of the CP-AMPARs is thought to allow for synaptic malleability, whereas CI-AMPARs contribute to basal neurotransmission and synaptic stability [22,92]. Consistent with this, when memory is retrieved, CI-AMPARs are transiently replaced by CP-AMPARs, triggering the destabilization process [64,98]. Notably, blocking either GluA2 endocytosis or protein degradation in the amygdala can prevent reconsolidation-dependent fear memory updating [64]. As blocking GluA2 endocytosis also prevented retrieval-induced increases in a marker of protein degradation, this work strongly suggests that GluA2 endocytosis is upstream of protein degradation.

Phosphorylation of the GluA1 subunit may also contribute to the stability of CP-AMPARs. Following retrieval, GluA1 subunits are phosphorylated at Ser845, triggering their insertion into CI-AMPARs in place of calcium-impermeable GluA2 subunits. With no GluA2 subunits, the receptors become CP-AMPARs, allowing the synapse to be plastic [72,80]. This subunit exchange is thought to be necessary to the destabilization process as blocking the GluA2 subunits results in inhibition of destabilization [36,42,64,92,98], while a general AMPA inhibitor does not consistently result in destabilization inhibition [99]. These complementary findings show the importance of CP-AMPARs in destabilization; when CI-AMPARs are present, the memory is stable, but when receptors convert to CP-AMPARs in response to new information, the memory is made labile [42,92].

NMDARs are also important to destabilization, as they play a key role in controlling the calcium influx necessary for associative plasticity. Multiple studies have disrupted the destabilization process by treating animals with NMDAR antagonists, such as AP5 [50,98,99], although one study found that NMDARs are important for the restabilization process [50]. As blocking the NMDA receptor typically disrupts the destabilization process, the memory is not labilized in response to the retrieval trial and instead persists in its original, stable state, without incorporating new information [24,50,68,83,99]. The timing of NMDAR antagonist treatment is important when trying to prevent destabilization. When NMDAR antagonists are injected before training, destabilization is typically blocked, whereas treating after training does not usually lead to impairment, supporting the idea that NMDARs are involved in destabilization, the initial step in the reconsolidation process [50,99]. 

Calcium/calmodulin-dependent kinase II (CaMKII) is a major protein in the brain that plays a role in many cellular functions and plays a key role in memory maintenance [122]. Cao and colleagues (2008) found that increasing αCaMKII expression during retrieval led to an erasure of short-term (one hour old), and remote (one month old) fear memories, but the process in which CaMKII disrupted destabilization was unknown [45]. Jarome and colleagues (2016) found that blocking CaMKII activity in vivo prevents memory destabilization by disrupting retrieval-induced increases in proteasome activity. As CaMKII is activated by calcium influx, it may function as an important connection between CP-AMPAR/NMDAR activation and the increased protein degradation needed to destabilize memory. In addition, when CaMKII is inhibited, proteasome activity is also decreased, suggesting that CaMKII is downstream of proteasome activity [70].

Finally, dopaminergic receptors have also been implicated in memory destabilization. Dopaminergic receptors are a group of five (called D_1_–D_5_) G-protein coupled receptors that play roles in many biological processes, including learning and memory (specifically receptors D_1_ and D_2_) [123]. To investigate the role of dopaminergic receptors in destabilization, Rossato and colleagues (2015) treated animals with an infusion of SCH23390 (an antagonist to D_1_/D_5_ dopaminergic receptors) either before or after an ORM update session to determine if the receptors were required for destabilization or restabilization, respectively. They found that blocking D_1_/D_5_ receptors before (but not after) the memory update prevented destabilization. This is consistent with the idea that D_1_/D_5_ dopaminergic receptors play a role in the initial destabilization phase but not in the subsequent restabilization phase [88].

### 4.2. Molecular Mechanisms of Memory Restabilization

The second phase of reconsolidation is restabilization, the process through which a destabilized memory is placed back into stable storage after incorporating the updated information. The most well-studied restabilization requirement is protein synthesis. Many groups have shown that inhibiting protein synthesis after reactivation leads to amnesia [19,26,44,61,62,66,87,94], presumably because new proteins are needed to restabilize the synapses after the new information is incorporated. In particular, translation through the mammalian target of rapamycin (mTOR) pathway, which regulates local protein synthesis within dendritic spines [124,125], is required for restabilization, suggesting that local protein translation within dendrites may play a critical role in reconsolidation-dependent memory updating. Notably, many studies have also shown that protein synthesis requirements can be situation-dependent, with boundary conditions (mentioned in Section 2) limiting whether protein synthesis inhibition can effectively disrupt the reconsolidation process. For example, remote memories that are over a month old are typically resistant to post-retrieval protein synthesis inhibition [37]. Likewise, especially strong memories are often not affected by protein synthesis inhibition [28,37]. Similarly, it is worth noting that other researchers have demonstrated that the amnesia produced by protein synthesis inhibition is not always permanent and memory can sometimes recover over time [51]. Overall, however, it is widely accepted that protein synthesis is critical for the restabilization phase.

It is less clear which individual proteins are needed to restabilize memory during a reconsolidation-dependent update, although many of the mechanisms needed for consolidation are also required for reconsolidation [15,120]. Lee and colleagues (2004), however, identified two proteins that play dissociable roles in consolidation and reconsolidation: BDNF and Zif268. These researchers found that BDNF is required for consolidation (but not reconsolidation), while Zif268 is required for reconsolidation (but not consolidation). This study was one of the first to demonstrate that consolidation and reconsolidation require unique mechanisms [76]. Although it is likely that other mechanisms are also uniquely engaged by reconsolidation-dependent memory updating, compared to the initial consolidation phase, the identity of these unique mechanisms is currently unknown.

In some cases, transcription also seems to be necessary for restabilization. For one, CREB, which regulates transcription, is required for restabilization. CREB activity is increased in the amygdala following fear memory retrieval [67] and blocking CREB in the amygdala impairs reconsolidation [49]. Further, Zif268, which plays a unique role in reconsolidation, as mentioned above, has a CREB-binding site, suggesting that CREB-mediated transcription may play a key role in restabilizing memories after updating. Consistent with this, blocking new mRNA synthesis in the amygdala can disrupt fear memory reconsolidation, suggesting that at least some of the necessary proteins are generated by de novo mRNAs, not mRNAs that were present prior to the restabilization process [62]. Notably, this may not be the case for all memories, as Parsons and colleagues demonstrated that protein, but not mRNA synthesis in the amygdala is required for fear memory restabilization [82]. Very little is currently understood about which updating circumstances require transcription and which individual genes might be required for reconsolidation-dependent updating.

Transcription is controlled, in part, by epigenetic mechanisms, which modulate gene expression by altering the chromatin structure, rather than changing the DNA sequence itself. Unsurprisingly, epigenetic mechanisms play a key role in the restabilization phase of reconsolidation [106]. Although there are only a few studies that manipulate epigenetic mechanisms during reconsolidation (for review, see Kwapis and Wood 2014 [126]), the results are largely consistent with evidence suggesting that epigenetic changes that facilitate transcription typically enhance restabilization whereas epigenetic mechanisms that restrict transcription usually impair restabilization. For example, blocking histone deacetylase (HDAC) activity, which typically opens the chromatin to permit transcription, can enhance reconsolidation [78,102]. In contrast, blocking histone acetyltransferase (HAT) activity, which typically creates a repressive chromatin structure, impairs reconsolidation [79]. As epigenetic mechanisms can have powerful and lasting effects within a cell, it will be important to better understand how individual chromatin modifiers contribute to reconsolidation-dependent memory updating and potentially modulate an organism’s response to subsequent memory updates.

## 5. Conclusions

Although the study of reconsolidation-dependent memory updating is still in the early stages, understanding how memories are modified with new information is an important area of research. In the clinic, reconsolidation-dependent memory updating could be leveraged to weaken maladaptive memories to treat PTSD, addiction [4] and emotional psychiatric disorders [3]. In the lab, understanding how memories are modified is also an important task, as existing memories needs to be constantly revised in order to remain relevant [1,2,3]. Much of what we understand about reconsolidation-dependent memory updating comes from the use of fear conditioning, a powerful system that has identified a number of molecular mechanisms that support reconsolidation-dependent memory updating. As this work expands into new behavioral paradigms that allow for novel and exciting comparisons, we hope that the molecular- and circuit-level mechanisms that support reconsolidation-dependent memory updating will become clear. Moving forward, it will be important to identify the molecular mechanisms that uniquely support reconsolidation-dependent memory updating (compared to initial memory consolidation). Further, work will need to clarify the roles of individual epigenetic mechanisms in the restabilization process. Finally, it will be important to understand how aging affects these mechanisms to impair the reconsolidation-dependent memory updating process. Identifying the mechanisms that support successful reconsolidation-dependent memory updating is the first step toward both modifying problematic memories and improving reconsolidation-dependent memory updating when it fails. 

## Figures and Tables

**Table 1:** Key reconsolidation-dependent updating studies from each behavioral paradigm covered in this review.

Paradigm	Pros	Cons	Sources
Fear Conditioning	Stimuli presentation controlled Number of pairings controlled Pairing timing controlled Behavior is robust, predictable, and measurable Cross-species Model for human diseases	Strong memory resistant to updating Older memories harder to update Unable to determine if reaction is to original or updated memory Cannot assess both memories in single session	[5,6,7,9,12,19,20,21,22,26,28,33,35,36,39,40,42,43,45,49,51,54,55,56,59,60,61,64,67,68,70,71,75,76,78,79,82,84,92,93,95,98,99,100,105,106]
Reconsolidation-Extinction	More permanent extinction Cross-species Model for treating human diseases	Does not persistently attenuate memories Remote memories more resistant	[46,47,48,52,53,58,69,72,73,74,77,80,91,107,108,109]
Morris Water Maze	Relies on hippocampus Well-studied spatial task	Stressful for animal Less appropriate for older animals Multiple potential confounding variables- stress hormones, exercise	[38,50,81,86,87,90,92]
Object Recognition Memory	Non-emotional and low stress Can look at original and updated memories independently	Animals must be in separate groups Relies on a less studied brain region, the perirhinal cortex	[44,45,88,89]
Objects in Updated Locations	Relies on hippocampus Can look at original and updated memories independently Non-emotional and low stress Able to identify age-related impairments	New, and therefore not as well-studied as other paradigms Only tested on mice	[2,41]

**Table 2:** Molecular mechanisms required for the destabilization and restabilization phases of reconsolidation-dependent memory updating.

Destabilization			
Process	Manipulation	Memory Effect	Sources
Protein degradation	Proteasome inhibitor	Prevented pharmacological-induced amnesia	[35,71,75]
CP-AMPARs	Pharmacological inhibition	Did not prevent pharmacological-induced amnesia	[99]
		Prevented updating	[31,42,92]
	Synthetic GluA2 causing inhibition		[36,98]
	Pharmacological inhibition of GluA2		[64]
	Performed multiple retrieval events	Affected GluA1 phosphorylation	[72,80]
	Pre-exposed animal to retrieval context to prevent memory updating	No increase in GluA2 subunits following retrieval session and induced amnesia	[22]
NMDARs	Pharmacological inhibition	Prevented updating	[24,50,63,68,83]
		Prevented pharmacological-induced amnesia	[99]
CaMKII	RNAi/Plasmid knockdown	Prevented updating	[29,43]
	Pharmacological inhibition		[29,70]
	Chemical-genetic overexpression		[45]
	Plasmid overexpression	High overexpression prevented updating Low overexpression did not prevent updating	[43]
Dopaminergic	Pharmacological inhibition	Prevented pharmacological-induced amnesia	[88]
**Restabilization**			
Protein synthesis	PKA pharmacological inhibition	Prevented updating	[93]
	Heat shock		[31]
	Pharmacological inhibition		[11,12,19,26,32,34,37,50,59,60,61,66,72,86,87,89,94,96,100,101]
		Prevented updating for a limited time	[51]
		Updating not inhibited due to boundary conditions	[28,37,101]
mRNA/Transcription	Fear conditioning	Increase in CREB transcription in amygdala and not the hippocampus	[67]
	mRNA pharmacological inhibitor	Did not prevent updating	[82]
		Prevented updating	[32,62,90]
	CREB transgenic mice		[49,50]
	Zif268 inhibition using ASO		[9,19,75,76]
	Zif268 mutant mice		[44]
Epigenetic mechanisms	HDAC2 pharmacological inhibition	Enhances reconsolidation	[78,102]
	HAT pharmacological inhibition	Impairs reconsolidation	[79]

## Abbreviations

AMPA	α-amino-3-hydroxy-5-methyl-4-isoxazolepropionic acid
AP5	(2R)-amino-5-phosphonovaleric acid
BDNF	Brain-derived neurotrophic factor
BLA	Basolateral nucleus of the amygdala
CaMKII	Calcium/calmodulin-dependent kinase II
CI-AMPARs	Calcium-impermeable AMPARs
CNQX	6-cyano-7-nitroquinoxaline-2,3-dione
CP-AMPARs	Calcium-permeable AMPA receptors
CPFE	Context pre-exposure facilitation effect
CR	Conditioned response
CREB	cAMP response element-binding protein
CS	Conditioned stimulus
DNQX	6,7-dinitroquinoxaline-2,3-dione
HAT	Histone acetyltransferase
HDAC	Histone deacetylase
mTOR	Mammalian target of rapamycin
MWM	Morris water maze
NMDA	N-Methyl-d-aspartic acid
ORM	Object recognition memory
OUL	Objects in updated locations
PTSD	Post-traumatic stress disorder
UCS	Unconditioned stimulus
Zif268	Zinc-finger 268 protein
